# Infrared Spectra of Hydrogen-Bonded Molecular Complexes Under Spatial Confinement

**DOI:** 10.3389/fchem.2021.801426

**Published:** 2022-01-07

**Authors:** Marta Chołuj, Josep M. Luis, Wojciech Bartkowiak, Robert Zaleśny

**Affiliations:** ^1^ Faculty of Chemistry, Wroclaw University of Science and Technology, Wrocław, Poland; ^2^ Institute of Computational Chemistry and Catalysis and Department of Chemistry, University of Girona, Catalonia, Spain

**Keywords:** infrared spectrum (IR), spatial confinement, harmonic oscillator potential, hydrogen-bonded complexes, vibrational transition intensity

## Abstract

Infrared (IR) spectroscopy is commonly used in chemical laboratories to study the geometrical structure of molecules and molecular complexes. The analysis of experimental IR spectra can nowadays be reliably supported by the results of quantum-chemical computations as vibrational frequencies and corresponding vibrational transition intensities are routinely calculated using harmonic approximation by virtually all quantum chemistry packages. In the present study we combine the methodology of computing vibrational spectra using high-level electron correlation treatments with an analytical potential-based approach to take into account spatial confinement effects. Using this approach, we perform a pioneering analysis of the impact of the spatial confinement caused by a cylindrical harmonic oscillator potential on the harmonic vibrational transition intensities and frequencies of two hydrogen-bonded complexes: HCN…HCN and HCN…HNC. The emphasis is put on the largest-intensity bands, which correspond to the stretching vibrations. The obtained results demonstrate that embedding the molecular complexes in an external confining potential causes significant changes of transition intensities and vibrational frequencies.

## 1 Introduction

Studies of molecular structure using infrared spectroscopy have become a fairly routine task and nowadays IR spectrometers are part of virtually all chemical laboratories. This technique (as well as other complementary vibrational spectroscopies) allows to gain an insight into the geometrical structure of molecules and molecular complexes. In fact, changes in frequencies and intensities of some key vibrational transitions are used as fingerprints to monitor the hydrogen or halogen bonds of molecular complexes. The analysis of experimental IR spectra can nowadays be reliably supported by the results of quantum-chemical computations as vibrational frequencies and corresponding transition intensities are routinely calculated using harmonic approximation by practically all packages. High-level electron-correlation methods can be used for band assignments in vibrational spectra of small- and medium-sized molecules and molecular complexes. Moreover, they can also be used to map the changes in infrared vibrational spectroscopic signatures to structural changes. The present study contributes to these theoretical efforts and its goal is to apply the methodology to compute the harmonic infrared vibrational spectra using high-level electron correlation treatments to spatially confined hydrogen-bonded molecular complexes.

The term “spatial confinement” is used throughout this work to refer to atoms and molecules trapped inside chemical cages (such as zeolites, mesoporous silica/organosilica, metal-organic frameworks, nanotubes and fullerenes), matter under high pressure, quantum wells, wires and dots as well as other constraining environments (Jaskólski (1996); Sen (2014)). On the theoretical basis, the spatial confinement is often simulated by external repulsive potentials, which reproduce the effect of orbital compression (connected with valence repulsion). This approach describes the interaction of objects with chemically and electronically inert environments (Jaskólski (1996)). Over the years, much effort has been devoted to the studies of the spatial confinement phenomenon, demonstrating its significant effects on the physical and chemical properties of atomic and molecular systems (Bartkowiak et al. (2021); Buchachenko (2001); Kozłowska et al. (2014); Chattaraj and Sarkar (2003); Cammi et al. (2008, 2012); Zaleśny et al. (2013); Góra et al. (2012); Lo and Klobukowski (2006)). Numerous interesting results were obtained, e.g., it was shown that the spatial confinement, in the form of analytical potential, causes an increase of the total energy (Buchachenko (2001); Bartkowiak and Strasburger (2010); Bielińska-Wa̧ż et al. (2001)) and the separation of HOMO and LUMO orbitals (Borgoo et al. (2008, 2009); Kozłowska et al. (2014)). Upon increasing the confinement strength, the polarizability of the spatially restricted systems decreases (Holka et al. (2005); Góra et al. (2012); Lo and Klobukowski (2006); Zaleśny et al. (2015); Kozłowska et al. (2015); Zaleśny et al. (2017)). Moreover, a shortening of the bond lengths was reported for molecules and molecular complexes embedded in confining potentials (Cammi et al. (2008, 2012); Zaleśny et al. (2013); Zaleśny et al. (2015)). Nevertheless, despite recent progress, there are still many aspects of spatial confinement which have not received sufficient attention yet. One of them is the modeling of IR spectra of spatially confined molecules and molecular complexes. In this context, one should not overlook the paper by Bonfim and Pilling who studied the effect of chemical environment on some properties of “trapped molecules” by using Polarizable Continuum Model (Bonfim and Pilling (2017)). These authors considered the effect of dielectric constant (3 < *ϵ* < 180) on vibrational frequencies and transition intensities. It was demonstrated that in the case of all analyzed molecules, i.e., CO, CO_2_, H_2_O—components of the astrophysical ices matrix, the increase of the value of dielectric constant is accompanied by larger band strengths. Other literature reports are largely limited to the analysis of the spatial confinement effect on shifts of vibrational frequencies (Shameema et al. (2006); Jana and Bandyopadhyay (2011); Song et al. (2005); Pagliai et al. (2014); Cammi et al. (2012); Boccalini et al. (2021)). Therefore, the goal of this project is to fill this gap by performing a pioneering analysis of the impact of spatial restriction, modeled by confining cylindrical analytical harmonic potential, on the infrared spectra (vibrational frequencies and transition intensities) of hydrogen-bonded molecular complexes.

## 2 Methods

In this work, the effect of spatial confinement on the vibrational properties of HCN…HCN and HCN…HNC complexes, with symmetry axis parallel to Cartesian *z* direction, was modeled by the cylindrical harmonic oscillator potential:

$$V_{conf}\left({\vec{r}}_{i}\right)=\frac{1}{2}\omega^{2}\left(x_{i}^{2}+y_{i}^{2}\right),$$ (1)

where the *ω* parameter controls the strength of spatial confinement, which is obtained by changing the curvature of the harmonic potential and the ${\vec{r}}_{i}$ refer *only* to the electronic coordinates. Thus, the effect of confinement on nuclei is accounted for indirectly by its effect on the electrons. Because of its symmetry, the cylindrical harmonic oscillator potential can be used as simplified representation of nanotube-like confining cages. *V*_*conf*_ as given by Eq. 1 was added to the Hamiltonian of the isolated complexes and subsequently vibrational-structure calculations were performed. In particular, the calculations of the infrared intensity for *i*-th mode (*I*_*i*_) under the influence of spatial confinement were performed based on the following equation:

$$I_{i}=\frac{N\pi }{3c^{2}}\underset{j=x,y,z}{\sum }{\left(\frac{\partial \mu_{j}}{\partial Q_{i}}\right)}^{2}$$ (2)

where *N* is Avogadro’s number, *c* is speed of light and $\frac{\partial \mu_{j}}{\partial Q_{i}}$ is derivative of *j*-th Cartesian component of dipole moment with respect to normal mode *Q*_*i*_.

Vibrational-structure computations were performed using MP2 and CCSD(T) methods and aug-cc-pVTZ basis set. MP2 method was used to determine gas-phase equilibrium geometries, vibrational frequencies and transition intensities analytically, as implemented in the GAUSSIAN 2009 package (Frisch et al. (2009)). On the other hand, at the CCSD(T)/aug-cc-pVTZ level the geometries of the studied complexes were optimized in vacuum and in the presence of cylindrical harmonic oscillator potential using custom computer routines based on total energies computed with the aid of the GAUSSIAN 2009 package (Frisch et al. (2009)). The calculations of infrared intensities of the spatially confined molecular complexes were performed at the CCSD(T)/aug-cc-pVTZ level using custom computer routines. To that end, we employed double numerical differentiation, i.e., the dipole moment components were computed numerically for a mesh of electric fields *F* (±2^*n*^ × *F*, where *n* = 0, …, 8, *F* = 0.000 2 au) at a set of displaced geometries (with mesh ±2^*m*^ ×Δ, where *m* = 0, …, 4, Δ = 0.01 bohr). The numerical accuracy of both numerical diffferentiation procedures was controlled with the aid of Romberg-Rutishauser scheme (Medved et al. (2007)).

## 3 Results and Discussion

To investigate the effect of spatial confinement on vibrational transition intensities, we chose two hydrogen-bonded complexes, i.e., HCN…HCN and HCN…HNC. The rationale behind selection of these two complexes stems from the results presented in the earlier studies by some of the present authors, devoted to the analysis of the effect of spatial confinement on the electronic and nuclear relaxation polarizability as well as first and second hyperpolarizability of molecules and molecular complexes (Zaleśny et al. (2015); Zaleśny et al. (2017)). The nuclear relaxation (hyper) polarizabilities are the major contribution to the vibrational (hyper) polarizabilities and include all their low-order corrections (Bishop (1998); Kirtman et al. (2000)). There are two main conclusions of these studies. Firstly, the effect of spatial confinement increases with the order of electrical property, both for electronic and vibrational counterparts, and it is much more pronounced for the former. In other words, the least significant effect was observed for electronic and nuclear relaxation polarizability. Secondly, the decrease of vibrational first hyperpolarizability was largely due to the harmonic part. The nuclear relaxation polarizability can be directly linked with infrared intensity given by Eq. 2 as both involve electric dipole moment (*μ*) derivatives with respect to normal modes (*Q*). Average nuclear relaxation polarizability is given by:

$$\langle \alpha^{\text{nr}}\rangle =\frac{1}{3}\underset{j=x,y,z}{\sum }\alpha_{jj}^{\text{nr}}=\frac{1}{3}\underset{i=1}{\sum }\frac{1}{\omega_{i}^{2}}\left[\underset{j=x,y,z}{\sum }{\left(\frac{\partial \mu_{j}}{\partial Q_{i}}\right)}^{2}\right]=\underset{i=1}{\sum }{\langle \alpha^{\text{nr}}\rangle }_{i}$$ (3)

where *i* labels normal modes with angular frequencies *ω*_*i*_. We may re-write the equation defining the nuclear relaxation polarizability in terms of the IR intensity *I*_*i*_:

$$\langle \alpha^{\text{nr}}\rangle =\underset{i}{\sum }{\langle \alpha^{\text{nr}}\rangle }_{i}=\frac{c^{2}}{N\pi }\underset{i}{\sum }\frac{I_{i}}{\omega_{i}^{2}}$$ (4)

Then for each mode we have the following relationship between the nuclear relaxation contribution to mode *i* and the corresponding intensity *I*_*i*_:

$$I_{i}∼\omega_{i}^{2}{\left\langle \alpha^{\text{nr}}\right\rangle }_{i}$$ (5)

It follows from the above relations that the effect of spatial confinement on nuclear relaxation polarizabilities can be predominantly manifested by the contributions from low-frequency normal modes, provided that corresponding dipole moment derivatives are significant. These expressions also allow for rationalizing why the absolute value of *α* ^nr^ is more than order of magnitude larger for HCN…HCN than for HCN (Zaleśny et al. (2015); Zaleśny et al. (2017)). In the case of hydrogen-bonded complexes there are X-H stretching modes corresponding to significant dipole moment changes which give rise to large-intensity bands. One may thus expect a significant effect of spatial confinement on vibrational intensities for such modes. Taken together, these evidences support the choice of HCN…HCN and HCN…HNC for the pioneering analysis performed in this study.

In what follows we will analyze the effect of confinement by choosing two values of *ω* parameter, i.e., 0.1 and 0.2 au. It was shown that these values approximately correspond to the realistic chemical environment, i.e., they reproduce exchange repulsion for linear weakly-bound complexes enclosed in carbon nanotubes (Zaleśny et al. (2017)). Such link can be established by comparison of the Hartree-Fock interaction energies between both studied complexes and the (4,4) carbon natotube with the results obtained using the analytic cylindrical confining potential. The results presented in Ref (Zaleśny et al. (2017)) demonstrate that the HF interaction energy between the (4,4) nanotube and HCN…HCN (HCN…HNC) is 133 kcal/mol (134 kcal/mol). The amplitude of the confining potential (*ω*) can be adjusted to represent this repulsive interaction:

$$\Delta E_{\text{int}}^{\text{HF}}\approx E\left(\omega \right)-E\left(\omega =0.0\right)$$

The amplitude *ω* = 0.1 a.u., through the above equation, roughly corresponds to Hartree-Fock interaction energy of HCN…HCN and HCN…HNC complexes trapped inside (4,4) carbon nanotube. The second chosen value of the amplitude thus corresponds to much larger confining environment caused by a carbon nanotube with smaller diameter.

**FIGURE 1:**
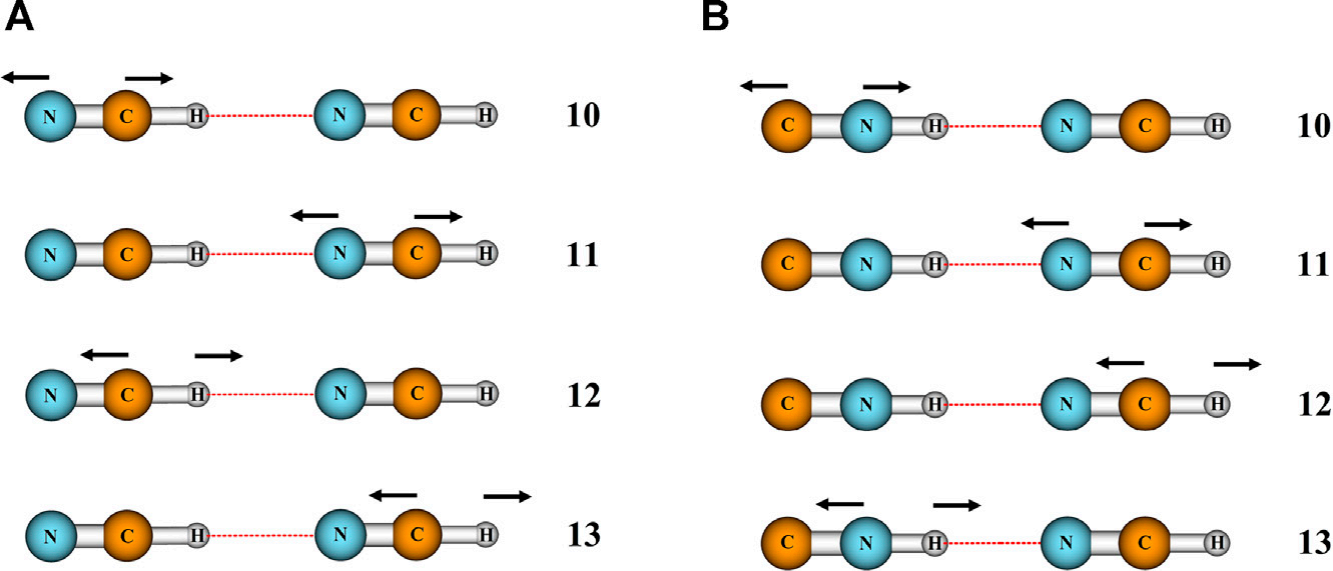
Stretching vibrations of HCN…HCN **(A)** and HCN…HNC **(B)** complexes and their labeling.

In order to shed light on the vibrational structure of isolated complexes, we evaluated the infrared intensities for all vibrational modes of HCN…HCN and HCN…HNC complexes, hereafter shortly referred to as (A(*i*)) and (B(*i*)). The results of calculations, performed at the cost-effective MP2/aug-cc-pVTZ level of theory, are presented in Table 1. Modes 12 and 13 have the largest IR intensities among all vibrations and this holds for both studied complexes. In particular, the IR intensities of the C-H and N-H stretching vibrations involving the hydrogen of hydrogen bond (A (12) and B (13)) are equal to 389 and 1,190 km/mol, respectively, and dominate the IR spectrum. In the reminder of the analysis, we will use highly accurate CCSD(T)/aug-cc-pVTZ level of theory to analyze the effect of confinement on four stretching modes presented in Figure 1, corresponding to the two C-N stretchings (A (10,11) and B (10,11)) and to the C-H (A (12,13), B (12)) and N-H stretchings vibrations (B (13)). We will start with the analysis of the influence of spatial confinement on the vibrational frequencies. Table 2 reports the computed vibrational frequencies of HCN…HCN and HCN…HNC complexes in vacuum and in the presence of confinement represented by the cylindrical harmonic oscillator potential. It is clear from these data that *ν*_*i*_ for modes 10-13 of both complexes are shifted to higher values by the spatial confinement, which agrees with the fact that the energy of a chemical systems increases under confinement. The increase of all *ν*_*i*_ values upon growing confinement strength from *ω* = 0.0 to *ω* = 0.1 au falls in between 33.7 and 56.1 cm^−1^. On the other hand, the changes caused by the confinement when its strength is equal to 0.2 au are much more pronounced, as the increase of *ν*_*i*_ ranges from 117.7 to 198.1 cm^−1^. Although the absolute shift of the frequencies caused by the spatial restriction is larger for the C-H and N-H stretchings (modes A (12,13) and B (12,13)) than the frequencies of the other two modes (A (10,11) and B (10,11)), analyzing the relative shift respect to the vacuum frequency is very similar for the four vibrations (i.e., 1.4–1.7% for *ω* = 0.1 au and 4.9–5.8% for *ω* = 0.2 au). Similar findings, i.e., an increase of vibrational frequencies under pressure, were reported for various molecular systems, such as diborane (Cammi et al. (2012)), C_60_ and C_70_ fullerenes (Pagliai et al. (2014)) and SF_6_ molecule (Boccalini et al. (2021)).

**TABLE 1:** Harmonic frequencies (*ν*_*i*_, in cm^−1^) and infrared intensities (*I*_*i*_, in km/mol) for vibrational normal modes *i* of HCN…HCN and HCN…HNC complexes computed at the MP2/aug-cc-pVTZ level of theory.

	HCN…HCN		HCN…HNC	
*i*	*ν*_*i*_	*I*_*i*_	*ν*_*i*_	*I*_*i*_
1	51.42	6.55	66.36	4.14
2	51.42	6.55	66.36	4.14
3	123.48	2.00	162.97	26.14
4	146.20	42.13	162.97	26.14
5	146.20	42.13	165.28	5.52
6	728.48	32.46	732.06	8.44
7	728.48	32.46	732.06	8.44
8	820.01	34.11	751.31	136.00
9	820.01	34.11	751.31	136.00
10	2019.47	8.86	2013.80	1.15
11	2038.53	0.97	2050.26	1.32
12	3380.56	389.39	3449.34	147.88
13	3452.89	76.78	3560.10	1190.32

**TABLE 2:** Frequencies (*ν*_*i*_, in cm^−1^) and infrared intensities (*I*_*i*_, in km/mol) for vibrational normal modes *i* of HCN…HCN and HCN…HNC complexes computed at the CCSD(T)/aug-cc-pVTZ level of theory.

*i*	*ν*_*i*_			*I*_*i*_		
	HCN…HCN					
	*ω* = 0.0 au	*ω* = 0.1 au	*ω* = 0.2 au	*ω* = 0.0 au	*ω* = 0.1 au	*ω* = 0.2 au
10	2103.1	2138.8	2225.1	15.10	15.45	16.54
11	2123.8	2157.9	2243.2	4.75	5.20	5.89
12	3363.8	3418.0	3556.9	346.96	336.32	311.94
13	3421.1	3477.2	3618.4	58.50	54.80	46.52
	**HCN…HNC**					
10	2044.7	2079.5	2166.4	11.80	11.14	9.86
11	2134.2	2167.9	2251.9	3.80	4.14	5.00
12	3418.8	3474.7	3616.9	109.16	102.52	89.37
13	3590.1	3641.1	3766.9	1119.50	1109.96	1098.96

We will now turn to the infrared intensities (see Table 2). Similarly to what we reported for vibrational frequencies, there is a monotonic perturbation in *I*_*i*_ for all four modes as the amplitude of confinement increases. The absolute changes in the IR intensities of modes A (10,11) and B (10,11) are very small and do not exceed 2 km/mol. Nevertheless, we highlight that for HCN…HCN the intensities of modes 10 and 11 *increase* on passing from *ω* = 0 through *ω* = 0.1 to *ω* = 0.2 au. The same is observed for mode 11 for HCN…HNC complex. In contrast, the modes A (12,13) and B (12,13) suffer a much stronger impact due to the spatial confinement, as there is a quite substantial decrease in *I*_*i*_ computed for unconfined and confined complexes that ranges from 3.7 to 20.54 km/mol. The largest-intensity bands in infrared spectra of the studied molecular complexes are the C-H and N-H stretchings which hydrogens are involved in the hydrogen bond (modes A (12) and B (13)). These two bands are the most significantly influenced by the spatial confinement. These observations can be further elucidated based on simple geometrical arguments. For *ω* = 0.1 a.u. there is shortening of all covalent bonds in HCN…HCN and HCN…HNC approximately by 0.006 Å, while the N…H bond length is shorter by 0.011 Å. Larger strength of confinement, i.e., *ω* = 0.2 a.u., leads to further shortening of covalent bonds (ranging from 0.020 to 0.022 Å with respect to the unconfined systems). Moreover, there is a significant decrease in the N…H bond length ranging from 0.035 Å (HCN…HCN) to 0.039 Å (HCN…HNC). The latter data explain the significant changes in intensities for modes A (12) and B (13). The confinement-induced geometrical changes observed for HCN…HCN and HCN…HNC complexes are in line with reports for other systems, e.g., CO_2_, C_2_H_2_, HCN and HCCCN (Zaleśny et al. (2013); Zaleśny et al. (2015)) Similar trends were also observed for the hydrogen bond length in other theoretical as well as experimental works concerning the effect of spatial confinement on the HB complexes (Lipkowski et al. (2014); Roztoczyńska et al. (2014); Ajami et al. (2011); Dougherty (1998)). As highlighted by Cammi et al. the bond shortening upon confinement can be linked with the deformation of the molecular electronic charge density, due to the Pauli repulsive interaction with the environment (Cammi et al. (2008; 2012)).

## 4 Summary

In summary, in the present study we demonstrated that the spatial confinement simulated by a cylindrical harmonic oscillator potential significantly affects vibrational spectra of hydrogen-bonded molecular complexes. Based on the obtained results we can draw several conclusions concerning the impact of spatial confinement on four stretching vibrations along molecular axis of HCN…HCN and HCN…HNC complexes:

• the changes in the vibrational transition intensities and corresponding frequencies are monotonic, and yet they are much more pronounced upon increasing the confinement strength from *ω* = 0.0 to *ω* = 0.2 au than on passing from *ω* = 0.0 to *ω* = 0.1 au;
• the presence of cylindrical harmonic oscillator potential leads to an increase of vibrational frequencies for all studied stretching vibrations;
• the vibrational transition intensities of modes corresponding to the C-H and N-H stretchings significantly decrease when the strength of the spatial confinement grows;
• the spatial confinement causes very small absolute changes in the IR intensities of C-N stretching vibrations and in the case of HCN…HCN complex the increase of confinement leads to increase in the corresponding vibrational transition intensities.

We believe that our results will not only contribute to broadening the knowledge on the spatial confinement phenomenon but they will also complement and support the experimental efforts in this field, by giving valuable insight into the nature of changes in the infrared spectra caused by spatial confinement. Our findings are particularly important from the point of view of astrochemistry, as the astrochemical measurements probe molecular species that experience extreme conditions, such as high pressure, and one of the most fundamental techniques used for exploration of cosmic space is the infrared spectroscopy. Finally, we highlight that in order to predict IR spectra of confined species with high accuracy it is mandatory to account for anharmonicity effects.

## Data Availability

The raw data supporting the conclusion of this article will be made available by the authors, without undue reservation.
